# Potent Anti-Inflammatory Effects of a Helix-to-Helix Peptide against *Pseudomonas aeruginosa* Endotoxin-Mediated Sepsis

**DOI:** 10.3390/antibiotics11111675

**Published:** 2022-11-21

**Authors:** Hyosuk Son, Seong-Cheol Park, Young-Min Kim, Jong-Kook Lee, Soyoung Park, Taeuk Guk, A-Mi Yoon, Hye Song Lim, Mi-Kyeong Jang, Jung Ro Lee

**Affiliations:** 1 Department of Chemical Engineering, Sunchon National University, Suncheon 57922, Republic of Korea; 2 Department of Exhibition and Education, National Marine Biodiversity Institute of Korea, Seocheon 33662, Republic of Korea; 3 LMO Team, National Institute of Ecology (NIE), Seocheon 33657, Republic of Korea; 4 Division of Life Sciences, Jeonbuk National University, Jeonju 54896, Republic of Korea

**Keywords:** antimicrobial peptide, septic shock, lipopolysaccharide, anti-inflammatory activity, cytokines

## Abstract

Although considerable scientific research data is available for sepsis and cytokine storm syndrome, there is a need to develop new treatments or drugs for sepsis management. Antimicrobial peptides (AMPs) possess anti-bacterial and anti-inflammatory activity, neutralizing toxins such as lipopolysaccharides (LPS, endotoxin). Most AMPs have been designed as a substitute for conventional antibiotics, which kill drug-resistant pathogens. The present study aimed to determine the anti-inflammatory potential of 10 designed XIW (X: lysine, arginine, or glutamic acid) α-helical peptides in macrophages and a mouse model in the presence of LPS. Among them, WIKE-14, a peptide with a helix-to-helix structure, having the 12th amino acid substituted with glutamic acid, suppressed pro-inflammatory cytokines in RAW 264.7 macrophages. This reaction was mediated by the inhibition of the binding between LPS and macrophages. In addition, the WIKE-14 peptide exhibited a potent anti-inflammatory activity in mice ears and lungs inflamed using LPS. Thus, our results may provide useful insights for the development of anti-sepsis agents via the sequence and structure information of the WIKE-14 peptide.

## 1. Introduction

Sepsis is one of the greatest threats to public health due to the emergence of drug-resistant bacteria and an increase in infection frequency worldwide [1,2,3]. Septic shock, an immune response to systemic bacterial infections, is primarily induced by endotoxins released from bacteria. A key endotoxin is lipopolysaccharide (LPS), the primary component of the outer membranes in gram-negative bacteria, consisting of core oligosaccharide (a structurally conserved hetero-oligosaccharide), lipid A (a phosphoglycolipid responsible for LPS toxicity, immunomodulation), and O-polysaccharide (an important surface antigen) [4,5]. Although LPS forms a physical barrier to protect bacteria from the surroundings, it is released into the blood stream or body fluid during bacterial death or division. It is subsequently complexed with lipopolysaccharide binding protein (LBP) and an inflammatory response of the host initiates the binding between LPS-LBP complex and Toll-like receptor 4 (TLR-4) in complex with myeloid differentiation factor 2 (MD-2) [6,7]. Tumor necrosis factor (TNF-α) and interleukin (IL)-1, IL-6, and IL-8 are a few examples of pro-inflammatory cytokines produced due to downstream signal activation. The excessive release of cytokines into the bloodstream can lead to multiple organ failure and eventually death [8,9,10]. Although considerable scientific research data is available for sepsis and cytokine storm syndrome, few drugs have been approved for the treatment of sepsis, with limited drug alternatives. Unfortunately, clinical trials have revealed the inefficiency of therapeutic approaches with anti-inflammatory cytokines [11]. Therefore, it is necessary to develop new treatments or drugs for sepsis management.

Antimicrobial peptides (AMPs) are emerging as new approaches to prevent and treat drug-resistant bacterial infections, and many studies are being conducted to evaluate their use as substitutes for conventional antibiotics [12,13,14]. AMPs are important components in defense mechanisms to attack pathogens via antimicrobial action and immunological responses such as chemotaxis and anti-endotoxic effects [15,16,17]. AMPs have a high affinity for LPS, mainly via electrostatic interactions, because gram-negative bacteria’s outer membrane contains several phosphate groups [18,19,20]. To elucidate the cell selectivity and action of AMPs, an understanding of LPS-AMP interaction is necessary.

The goal of this study was to look at the anti-inflammatory effects and anti-inflammatory mechanism of 10 designed AMPs, composed of Lysine (Lys) (substituted by arginine (Arg) or glutamic acid (Glu)), phenylalanine (Ile), and tryptophan (Trp) amino acids, in LPS-stimulated macrophages in vitro. Among them, a potent anti-inflammatory activity of WIKE-14 peptide, having a helix-to-helix structure, was assessed through an LPS-induced ear edema and acute lung injury (ALI) in a mouse model. Our findings suggested that the designed peptides can be developed as potent antibacterial agents, and one of them can potentially be used as a drug with antibacterial and anti-inflammatory activities.

## 2. Results and Discussion

### 2.1. Design and Synthesis of α-Helical Peptides

To develop highly efficient antibiotics and anti-inflammatory agents, it is necessary to have cell selectivity to exhibit potent activity and non-toxic properties. Teichoic acids in the peptidoglycan layer of gram-positive bacteria and LPS in the outer membranes of gram-negative bacteria are negatively charged. In addition, cytoplasmic membranes of bacteria contain phosphatidylglycerol (PG) and cardiolipin (CL) with an anionic polar head [21,22], whereas mammalian cells contain phosphatidylcholine and sphingomyelin with zwitterionic polar head, and cholesterol with hydrophobic body in the outer leaflet of the plasma membrane [23]. Therefore, the bacterial surface is negatively charged and the surface of mammalian cells is zwitterionic. The mechanism of anti-inflammatory activity is via direct action with external substances such as LPS and mediation of signal transduction in immune cells.

**Figure 1:**
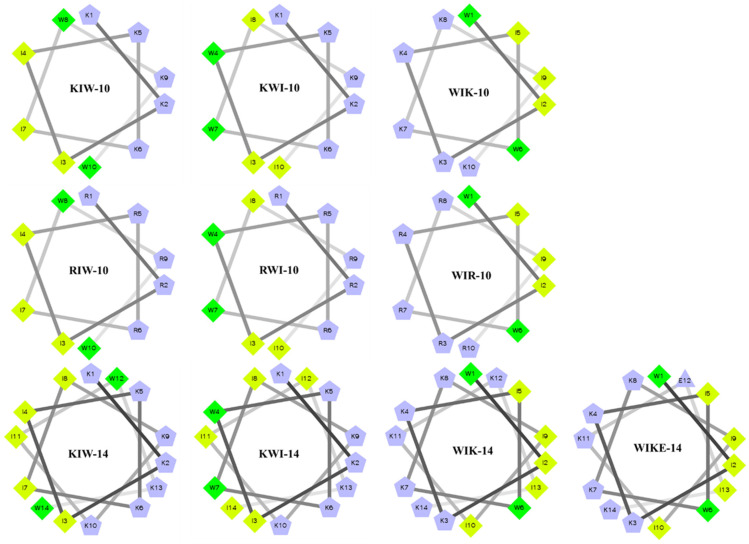
Helical wheel projections of de novo designed peptides with α-helical structure.

The peptides used in this study were designed to identify the sequence and structure of peptides that would possess antibacterial activity through membranolytic action and anti-inflammatory activity through LPS-neutralizing action. Lysine (Lys) or arginine (Arg) was used to attach with LPS as a cationic amino acid, and isoleucine (Ile) and tryptophan (W) were used to interact with lipid A of LPS as hydrophobic amino acids. All of the designed peptides have amphipathic structures, and two tryptophan residues are located between hydrophilic and hydrophobic interfaces or at the center of the hydrophobic region. When two tryptophan residues are located in the middle of the peptide sequence, they are present in the middle of the hydrophobic face in the wheel diagram (Figure 1). To reduce the strength of the positive charge, the 12th Lys of WIK-14 was replaced with glutamic acid (E).

### 2.2. Characterization, Antimicrobial Activity, and Cytotoxicity of XIW Peptides

Table 1 shows sequences, physicochemical characteristics, antimicrobial activities, and the cytotoxicity of 10 designed peptides, six peptides with 10 amino acids (10-mer) and four peptides with 14 amino acids (14-mer). The calculated hydrophobicity of the 10-mer peptides was higher than that of the 14-mer peptides. Chen et al., suggested that the hydrophobicity of a peptide correlates with peptide helicity and self-associating ability in aqueous environments [24]. They also suggested that the decreased antimicrobial activity of peptides with high hydrophobicity is due to their strong self-association, which inhibits their access to eukaryotic membranes [24]. Mean hydrophobic moment is a measure of the amphiphilicity of peptides in an alpha-helical structure, and a high value indicates that the helix is amphiphilic and perpendicular to its axis [25]. The geometric mean values of minimum inhibitory concentrations (GM) for the four bacteria were the lowest in the 14-mer peptides, and the 10-mer peptides were non-toxic in Raw 264.7 cells.

### 2.3. Secondary Structure of XIW Peptides in Aqueous Solutions

**Figure 2:**
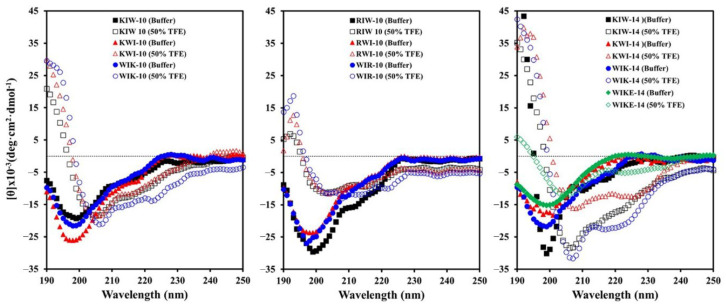
Secondary structures of XIW peptides in buffer and 50% TFE solutions. Peptides (50 µM) suspended in 10 mM sodium phosphate buffer (pH 7.2) and 50% TFE solutions were analyzed using a circular dichroism (CD) spectrometer.

The circular dichroism (CD) spectra of the designed peptides in 10 mM sodium phosphate buffer (pH 7.2) and 50% (*v*/*v*) 2,2,2-trifluoroethanol (TFE) solutions are shown in Figure 2. The spectra for all the peptides showed a high magnitude negative band at approximately 200 nm, indicating random coil structures. Structure analyses showed that TFE induces the increased population of α-helix and β-sheet content in secondary-structure-forming peptides in TFE/water mixtures [26,27]. In TFE solution, the two negative bands at 208 nm and 222 nm of all peptides indicate their ability to form a helical structure, though their helicity is different. In particular, compared to peptides with Lys, those substituted with Arg have a lower helicity and a loose structure. It was observed that WIKE-14 can exist in both helix and unraveled structures. In the secondary structure analysis of peptides, WIKE-14 was expected to have a helix-to-helix structure, whereas others have an α-helical structure.

### 2.4. Interaction of XIW Peptides with LPS

**Figure 3:**
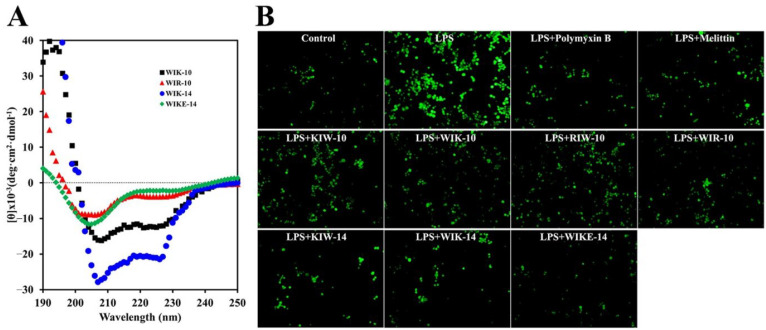
CD spectra of peptides in LPS and effects of peptides on reactive oxygen species (ROS) production. (**A**) Secondary structures of WIK-10, WIR-10, WIK-14, and WIKE-14 peptides (50 µM) in 1 µg/mL *Pseudomonas aeruginosa* LPS. (**B**) RAW 264.7 cells were pre-incubated in the absence or presence of peptides (8 µM) for 30 min and treated with or without LPS (100 ng/mL). After 24 h incubation, the cells were treated using H_2_DCFDA and incubated for 10 min, following which the fluorescent cells were observed under a fluorescence microscope.

CD spectra were analyzed to ascertain the secondary structures of WIK-10, WIR-10, WIK-14, and WIKE-14 in the LPS solution. As shown in Figure 3A, all peptides formed a helical structure even in 50% TFE. This suggests that the tested peptides can bind to LPS via electrostatic attraction.

Excess reactive oxygen species (ROS) production during various infections can activate inflammation via the mitogen-activated protein kinase signal transduction, resulting in the upregulation of inflammatory cytokines [28,29,30]. H_2_DCFDA, a fluorescent dye, was used to investigate the inhibitory action of peptides in LPS-stimulated ROS generation in RAW 264.7 cells. In this assay, polymyxin B, a cyclic peptide antibiotic which can neutralize endotoxins via its amine groups, was used as a control drug. LPS-treated macrophages produced a high level of ROS, but ROS-producing level of cells in the presence of 14-mer peptides (KIW-14, WIK-14, and WIKE-14) was significantly reduced, being similar to cells with non-treated LPS, as well as polymyxin B and melittin (Figure 3B). These results indicate that our peptides can protect LPS-induced ROS stimulation via the direct binding with LPS.

### 2.5. Inhibition of LPS Binding to RAW 264.7 Cells

**Figure 4:**
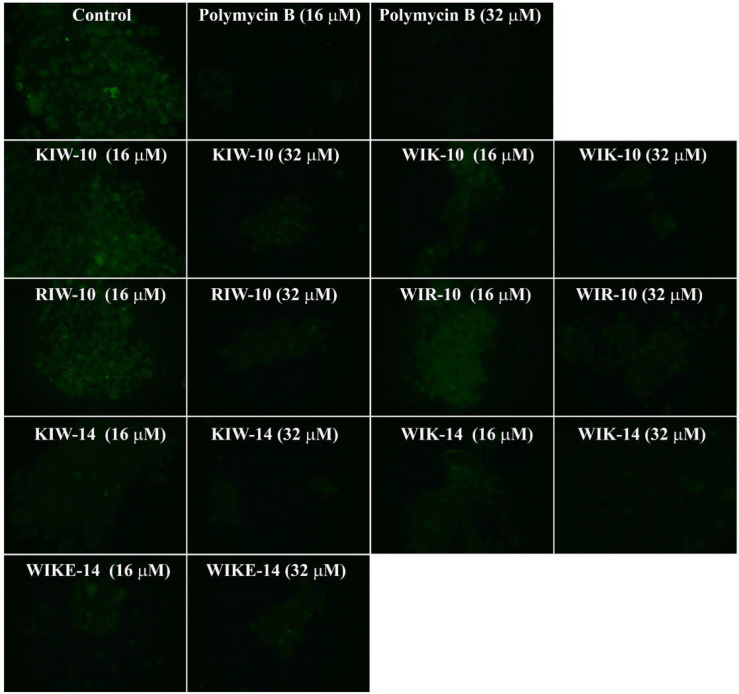
Inhibitory effects of peptides on binding between LPS and RAW 264.7 cells. Fluorescein isothiocyanate-labeled LPS and peptides were incubated for 12 h with RAW 264.7 cells, and cells were washed with PBS, followed by observation using fluorescence microscopy.

To examine how peptides inhibit the LPS-induced macrophage stimulation, fluorescein isothiocyanate (FITC)-LPS and individual peptides were pre-incubated, and the mixtures were then added to RAW 264.7 cells (Figure 4). In the presence of the peptide variants or polymyxin B, FITC fluorescence signals in RAW 264.7 cells dramatically decreased in a dose-dependent manner. In contrast, the LPS-treated cells without any peptides showed strong fluorescence emission.

**Figure 5:**
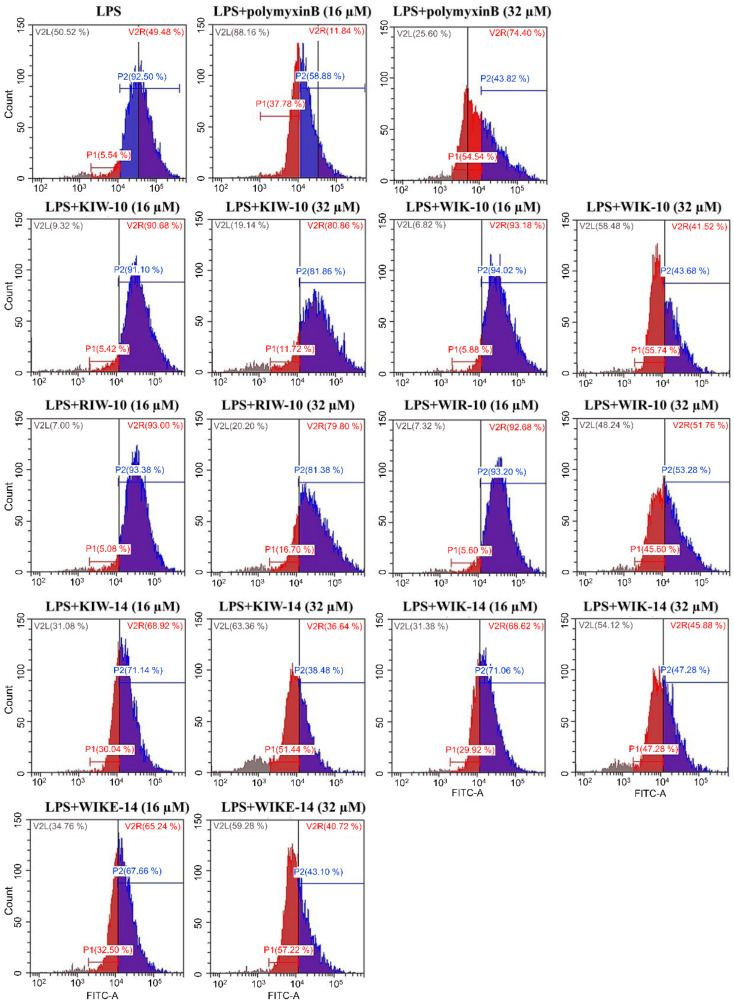
Inhibitory effects of peptides on the binding between LPS and RAW 264.7 cells. FITC-labeled LPS and peptides were incubated for 12 h with RAW 264.7 cells, and cells were washed with PBS, followed by measurement using flow cytometry.

Fluorescence-activated cell sorting (FACS) analysis of FITC-labeled LPS was applied to compare the effect of polymyxin B and the synthesized peptide variants in RAW 264.7 cells. In the absence of peptides, the percentage of FITC-positive cells was high, indicating the highly bound LPS on macrophage cells. However, treatment with the peptides (16 and 32 µM) decreased the percentage of LPS-binding with the macrophage cells in a concentration-dependent manner (Figure 5).

The results suggest that the synthesized peptides prevent the binding of LPS to macrophages. The cationic groups of the synthesized peptides could initially interact with the LPS phosphate residues through electrostatic interactions, and the hydrophobic residues of the peptides further completed the peptide-LPS complex. This resulted in a decrease in free LPS, and macrophages were not stimulated.

### 2.6. Inhibition of Nitric Oxide and Cytokine Production

**Figure 6:**
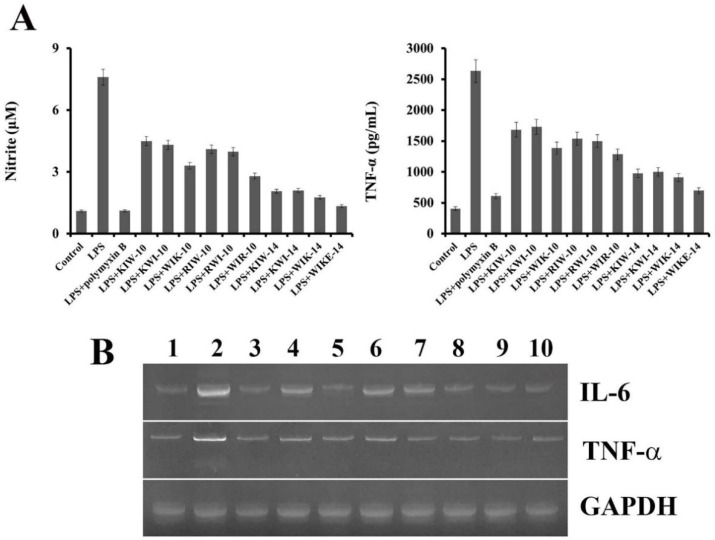
Anti-inflammatory effects of peptides in RAW 264.7 cells. (**A**) Secreted nitric oxide and tumor necrosis factor-α were measured from the supernatants after 24 h of LPS-stimulation with peptides (32 µM). (**B**) mRNA expression of RAW 264.7 cells with peptides. IL-6, interleukin-6, TNF- α, tumor necrosis factor-α; GAPDH, Glyceraldehyde 3-phosphate dehydrogenase. 1: control, 2: LPS, 3: LPS+Polymyxin B, 4: LPS+KIW-10, 5: LPS+WIK-10, 6: LPS+RIW-10, 7: LPS+WIR-10, 8: LPS+KIW-14, 9: LPS+WIK-14, 10: LPS+WIKE-14.

AMPs can counteract the endotoxin-induced nitric oxide (NO) and cytokine release, either by direct binding to LPS or by hindering the interaction between LPS and LBP. Quantitative levels of NO and TNF-α, a pro-inflammatory cytokine, secreted from the murine macrophage, RAW 264.7 cells, were evaluated to determine the anti-inflammatory effect of each synthesized peptide. As shown in Figure 6A, compared to the control (no treatment), LPS stimulation showed significant production of NO. In contrast, LPS-induced NO secretion in the presence of the peptide variants or an antibiotic (polymyxin B) was inhibited, indicating that the counteraction of LPS-induced NO production by the peptides. Notably, four peptides with 14-mer length more strongly inhibited NO secretion in RAW 264.7 cells than that by the remaining six peptide variants with 10-mer length. The TNF-α levels were also measured using an enzyme-linked immunosorbent assay kit in the LPS-stimulated cell supernatants in the presence of the peptide variants. Figure 6B shows that the peptides reduced the level of TNF-α secretion in a length-dependent manner.

Inflammatory cytokines, such as IL-6 and TNF-α, are essential factors in LPS-stimulated inflammation. Inhibition of NO and TNF-α release by the synthesized peptides was re-confirmed using mRNA expression level analysis of inflammation-related genes, of IL-6 and TNF-α in RAW 264.7 cells (Figure 6B). LPS-induced NO or TNF-α secretion in the presence of the peptides and an antibiotic (polymyxin B) was inhibited, and expression levels of IL-6 and TNF-α were clearly decreased compared to peptide-untreated cells (only LPS-treated cells), indicating the inhibition of LPS-induced inflammation by the peptides (Figure 6B).

### 2.7. In Vivo Anti-Inflammatory Effects of Peptides

**Figure 7:**
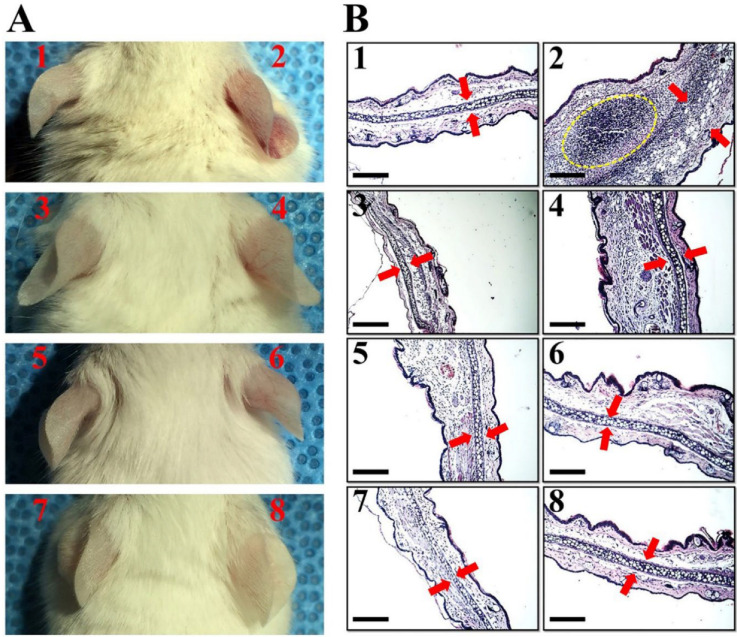
Anti-inflammatory effects of peptides on LPS-induced ear edema. (**A**) The ears of Balb/c mice (*n* = 3) were injected intradermally with LPS solution (0.02 µg). After 1 h, the indicated peptides were injected in 32 µM, followed by further incubation for 3 days. (**B**) Ear morphologies were digitally recorded, and collected ears were stained using hematoxylin and eosin (H&E) on paraffinized tissues. 1: phosphate buffered saline, 2: LPS, 3: WIK-10, 4: LPS+WIK-10, 5: WIK-14, 6: LPS+WIK-14, 7: WIKE-14, 8: LPS+WIKE-14. Scale Bar corresponds to 1 mm.

The antibacterial and anti-inflammatory activity of peptides in which Trp is located at the amino-terminal region is excellent compared to other peptides. Therefore, in order to compare the effect of peptide length and structure on anti-inflammatory activity in vivo, WIK-10, WIK-14 and WIKE-14 peptides were chosen for further study. To assess the in vivo anti-inflammatory effects of them, ear edema was induced in mice using LPS and peptides were injected intradermally in mice ears to the LPS-injected site. After 3 days, redness, swelling, and increased thickness were observed in the LPS-treated ears, compared with phosphate buffered saline (PBS)-injected ears (Figure 7A). Hematoxylin and eosin (H&E) staining of the tissues showed significantly increased neutrophils and infiltrated immune cells (yellow dotted circle in Figure 7(B2)), and disruption of cartilage layer (red arrow in Figure 7B) in the LPS-treated ears, indicating severe inflammation induction (Figure 7(B2)). Only peptides were injected into the ears to determine their ability to cause toxicity and inflammation in mice skin; however, there were no edema symptoms in both optical and histological analyses (Figure 7B). In the presence of WIK-10 and WIK-14 peptides, LPS induced slight inflammatory responses; however, WIKE-14 injected to mice ears treated with LPS showed no symptoms of edema or immune response (Figure 7(B8)).

**Figure 8:**
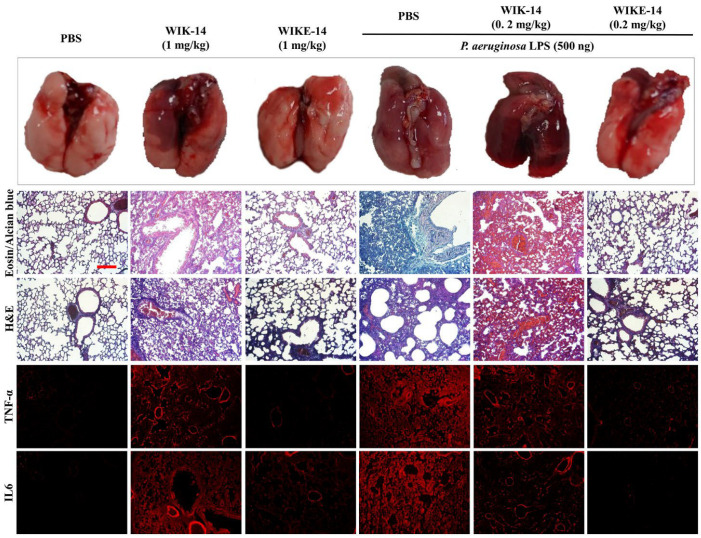
In vivo anti-inflammatory effects of peptides in acute lung injury. *Pseudomonas aeruginosa* LPS (500 ng) was intratracheally instilled in mice lungs. One hour after instillation, peptides were intratracheally administered, followed by monitoring for 3 days. Lungs collected from the euthanized mice were digitally visualized and tissues were stained using eosin/alcian blue, H&E, PE-conjugated TNF-α, and IL6 antibody. Bar is 50 µm.

To assess the anti-inflammatory action of peptides in acute lung injury (ALI), LPS was intratracheally instilled into mouse lungs. Morphological observation showed that LPS induced severe lung injury (Figure 8). Histological and immunological stains indicated increased mucin materials and neutrophilic infiltration, edema, fibrosis, and up-regulated cytokines. Figure 8 shows that WIK-14 (1 mg/kg) was toxic in lung tissue cells, but WIKE-14 (1 mg/kg) was non-toxic without any inflammatory responses. Interestingly, LPS-induced ALI was completely prevented by the instillation of WIKE-14 (0.2 mg/kg). We propose that WIKE-14 peptide exhibits a potent activity in the LPS-induced inflammation, owing to its helix-to-helix structure. In general, the cationic residue of the peptide binds to the phosphate groups in LPS, and the lipid A regions are strongly bound by the hydrophobic residues. At this point, the peptides with the helix-to-helix structure have a wide binding surface area with LPS due to the flexible region, which can be rotated to bind several LPS molecules.

## 3. Materials and Methods

### 3.1. Materials

Oxyma pure and 9-Fluorenylmethoxycarbonyl (Fmoc) amino acids were obtained from CEM Co. (Matthews, NC, USA). *Pseudomonas aeruginosa* LPS and TFE were purchased from Sigma-Aldrich Co. (St. Louis, MO, USA). Phycoerythrin-conjugated anti-IL-6 and anti-TNF-α were provided from BioLegend (San Diego, CA, USA). H_2_DCFDA was procured from Molecular Probes (Eugene, OR, USA). All other chemicals and solvents used were of analytical or reagent grade and were used as received.

### 3.2. Peptide Preparation

All peptides were synthesized using solid-phase methods with Fmoc-protected amino acids on a Liberty Microwave Peptide synthesizer (CEM Co., Matthews, NC, USA). Synthesis procedures were according to previously reported methods [31]. The synthesis yield and purity of all peptides used in this study was more than 85% and 98%, respectively.

### 3.3. Antibacterial and Cytotoxic Assay In Vitro

The antibacterial activity was examined using a microdilution assay with *Eschrichia coli* (ATCC 25922), *P. aeruginosa* (ATCC 15692) and *Staphylococcus aureus* (ATCC 25923), which were obtained from the American Type Culture Collection. *Listeria monocytogenes* (KCTC 3710) was obtained from the Korean Collection for Type Cultures. Cell survival was investigated using the 3-(4,5-dimethylthiazol-2-yl)-2,5-diphenyltetrazolium bromide assay in RAW264.7 cells. The assay was performed as described previously [32,33].

### 3.4. CD Analysis

CD spectra were collected at 25 °C on a Jasco-810 spectropolarimeter (Jasco, MD, USA). A quartz cell with 1 mm path-length was used with a 50 μM peptide solution and 10 mM sodium phosphate buffer (pH 7.2), 50% TFE co-solvent, or 1 µg/mL LPS solution. Three scans from 190 to 250 nm were acquired for each condition and averaged to improve the signal-to-noise ratio. Mean residue ellipticities ([*θ*], deg·cm^2^dmol^−1^) were calculated using Equation (1):

[*θ*] = *θ*_obs_/10 · *l* · *c* (1)

where *θ*_obs_ is the measured signal (ellipticity) in millidegrees, *l* is the optical path-length of the cell in cm, and *c* is the concentration of peptide in mol/L [mean residue molar concentration: *c* = number of residues in the constructed peptide × the molar concentration of the peptide] [33].

### 3.5. ROS Measurement

RAW 264.7 cells (1 × 10^6^ cells/mL) plated in a 24-well plate were pre-cultured for 24 h. Peptides in RPMI-1640 medium were then pretreated to cells for 30 min and *P. aeruginosa* LPS (100 ng/mL) was added, followed by additional incubation for 24 h. Control cells are not treated with peptides and LPS. H_2_DCFDA (10 µL, final concentration 10 µM) was treated to the cells, followed by incubation for 30 min in a CO_2_ incubator. The cells washed with cold-PBS buffer were then observed using a fluorescence microscope (OPTINIT KCS3-160S; Korea Lab Tech, Seongnam, Gyeonggi-do, Republic of Korea).

### 3.6. Inhibition of LPS Binding to RAW 264.7 Cells

RAW 264.7 cells cultured in Roswell Park Memorial Institute Medium (RPMI)-1640 containing 10% fetal bovine serum (FBS) were aliquoted to 1 × 10^6^ cells/mL in a 24-well plate, and cultured at 37 °C, 5% CO_2_ incubator for 24 h. Each well was treated with 1 µg/mL of FITC-attached LPS. After 1 h, peptides were added to the wells at the indicated concentrations. After incubation for 8 h, the cells were washed with cold-PBS, observed under a fluorescence microscope, and measured using flow cytometry (Beckman Coulter CytoFlex, Brea, CA, USA).

### 3.7. Nitric Oxide and TNF-α Production in LPS-Stimulated RAW 264.7 Cells

The murine macrophage cell RAW 264.7 was grown RPMI-1640 medium supplemented with 10% FBS and then plated at a density of 1 × 10^6^ cells/mL in a 24-well microplate. After incubation for 24 h, the cells were stimulated with *P. aeruginosa* LPS (1 µg/mL), followed by further incubation for 1 h. Peptides were incubated for 24 h. Nitrite was measured by adding 100 µL of cell culture extract to 100 µL of Griess reagent (1% sulfanilamide and 0.1% naphthylenediamine in 5% phosphoric acid). The nitrite concentrations were calculated by comparing the absorbance of sodium nitrite standard solutions. The optical density was evaluated at 550 nm in a microplate reader (Molecular Devices, Emax, CA, USA). TNF-α levels in culture supernatants were analyzed using the R&D system TNF-α EASIA kit, Minneapolis, MN, USA). The developed color was determined by measuring the OD at 490 nm in a microplate reader.

### 3.8. Cytokine mRNA Production from RAW 264.7 Stimulated by LPS

RAW 264.7 cells were cultured in RPMI-1640 medium containing 10% FBS, and then seeded to 5 × 10^6^ cells/mL in a 6-well plate and cultured at 37 °C, 5% CO_2_ incubator for 24 h. Each well was treated with 1 μg/mL of LPS; after 1 h, 32 μM of each peptide was added and incubated for 8 h. Cells were collected, and total RNA was isolated using TRIzol reagent (Thermo Fisher Scientific, Waltham, MA, USA). 5′-GCATCTTCTTGTGCAGTGCC-3′ and 5′-TCACACCCATCACAAACATG-3′ as Glyceraldehyde 3-phosphate dehydrogenase primers, 5′-CACAAGTCCGGAGAGGAGAC-3′ and 5′-CAGAATTGCCATTGCACAAC-3′ as IL-6 primers, and 5′-CGCAGCAGCACATCAACAAGAGC-3′ and 5′-TGTCCTCATCCTGGAAGGTCCACG-3′ as TNF-α primers were used for reverse transcription-polymerase chain reaction (RT-PCR). The PCR products generated using the Maxime RT-PCR premix kit (IntronBio, Seongnam, Gyeonggi-do, Republic of Korea) were confirmed using electrophoresis.

### 3.9. In Vivo Anti-Inflammatory Experiments

All animal experiments and processes were fulfilled with the approval of the Institutional Animal Care and Use Committee (IACUC) of Sunchon National University, Republic of Korea (SCNU IACUC-2019-10). Seven-week-old female Balb/c mice (Koatech Co., Pyongtaek, Gyeonggi-do, Republic of Korea) were anesthetized by inhalation of 5% (induction) and 2% (maintenance) isoflurane in pure oxygen and LPS (0.02 µg) was intradermally injected into the mice ears. After 1 h, the indicated peptides (32 µM) were injected, followed by further incubation for 3 days. The ears were digitally examined and collected for staining using hematoxylin and eosin (H&E). Histological observations using H&E staining were performed according to the following processes.

*P. aeruginosa* LPS (500 ng) in 50 µL PBS was intratracheally instilled into seven-week-old female Balb/c mice. One hour after exposure, each peptide was intratracheally administered at the indicated dose in 50 µL PBS. Control mice were injected with 50 µL PBS without the peptides. Mice were monitored for signs of morbidity for 3 days after LPS exposure. Mice were euthanized using CO_2_ inhalation, and the lung tissues were excised and fixed in 4% paraformaldehyde. The fixed tissues were dehydrated using a series of ethanol solutions (50−100%) and embedded in paraffin. The tissues embedded with paraffin were sectioned with a thickness of 5 µm (Leica microtome, Deerfield, MA, USA). Histopathology of the lung tissues was performed using alcian blue and eosin and H&E staining. An immunohistology was performed using monoclonal antibodies of PE-conjugated anti-IL-6 and anti-TNF-α (BioLegend, San Diego, CA, USA). The stained tissue sections were observed under a fluorescence microscope (OPTINIT KCS3-160S; Korea Lab Tech, Seongnam, Gyeonggi-do, Republic of Korea).

### 3.10. Statistical Data Analysis

The mean values of at least four independent determinations ± SD (Student *t*-test) were calculated using Excel software (Microsoft Office 2016).

## 4. Conclusions

In summary, the study findings suggest that all peptides inhibited the expression of LPS-induced NO and cytokines in RAW 264.7 cells by preventing the interaction between LPS and macrophages, though there are differences in their inhibitory efficiency. In addition, the WIKE-14 peptide with glutamic acid exhibited a more effective anti-inflammatory action than that observed in peptides without glutamic acid. Thus, the WIK-14 peptide can be developed as a potential antibacterial agent within the minimum inhibitory concentration, and the WIKE-14 peptide can potentially be used as a drug with both antibacterial and anti-inflammatory activities.

## Figures and Tables

**Table 1:** Amino acid sequences, hydrophobicity (*H*), hydrophobic moment (*µH*), antimicrobial activity, and cytotoxicity of XIW peptides.

Name	Sequence	*H* ^a^	*µH* ^b^	Net Charge	GM (µM) ^c^	Cytotoxicity (%) ^d^
KIW-10	KKIIKKIWKW-NH_2_	0.495	0.822	+6	11 ± 1	0 ± 0
KWI-10	KKIWKKWIKI-NH_2_	0.495	0.880	+6	10.5 ± 1.2	0 ± 0
WIK-10	WIKKIWKKIK-NH_2_	0.495	0.902	+6	6.5 ± 0.8	0 ± 0
RIW-10	RRIIRRIWRW-NH_2_	0.485	0.829	+6	8.5 ± 0.8	0 ± 0
RWI-10	RRIWRRWIRI-NH_2_	0.485	0.887	+6	7 ± 1.2	0 ± 0
WIR-10	WIRRIWRRIR-NH_2_	0.485	0.907	+6	3 ± 0.2	0.8 ± 0.2
KIW-14	KKIIKKIIKKIWKW-NH_2_	0.469	0.782	+8	2.6 ± 1.2	3.2 ± 0.8
KWI-14	KKIWKKWIKKIIKI-NH_2_	0.469	0.827	+8	2.6 ± 0.8	1.2 ± 0.4
WIK-14	WIKKIWKKIIKKIK-NH_2_	0.469	0.816	+8	2.1 ± 0.4	7.8 ± 1.2
WIKE-14	WIKKIWKKIIKEIK-NH_2_	0.494	0.823	+6	8 ± 0.8	0.3 ± 0.1

^a^ Mean hydrophobicity (*H*) and ^b^ mean relative hydrophobic moments (*µH*) were calculated in a HeliQuest Web server (http://heliquest.ipmc.cnrs.fr, accessed on 29 October 2022). ^c^ The geometric mean of minimum inhibitory concentration values against *Escherichia coli*, *Pseudomonas aeruginosa*, *Staphylococcus aureus*, and *Listeria monocytogenes*. ^d^ Cytotoxicity was assayed against RAW264.7 cells at a 100 µM peptide concentration.

## Data Availability

Not applicable.
